# Patient preferences for incidental findings in a genomic trial: Insights from the Copenhagen glioblastoma cohort

**DOI:** 10.1093/noajnl/vdag062

**Published:** 2026-03-22

**Authors:** Anders Overby Sjøstrøm, Maria Camilla Mathiasen, Ulrik Lassen, Karin Piil, Dorte Schou Nørøxe

**Affiliations:** Department of Oncology, University Hospital of Copenhagen, Rigshospitalet, Copenhagen, Denmark; Department of Oncology, University Hospital of Copenhagen, Rigshospitalet, Copenhagen, Denmark; Department of Oncology, University Hospital of Copenhagen, Rigshospitalet, Copenhagen, Denmark; Danish Comprehensive Cancer Center. Brain Tumor Center (DCCC. BTC), Copenhagen, Denmark; Department of Oncology, University Hospital of Copenhagen, Rigshospitalet, Copenhagen, Denmark; Faculty of Health and Medical Sciences, Department of Clinical Medicine, Copenhagen University, Copenhagen, Denmark; School of Nursing, Faculty of Health Sciences, Curtin University, Perth, Western Australia, Australia; Department of Oncology, University Hospital of Copenhagen, Rigshospitalet, Copenhagen, Denmark; Danish Comprehensive Cancer Center. Brain Tumor Center (DCCC. BTC), Copenhagen, Denmark

## Abstract

Glioblastoma (GBM) patients often face cognitive impairment, which may limit their capacity to comprehend complex trial information, particularly regarding genomic analyses and incidental findings. We analyzed consent forms from a genomic study, including 108 newly diagnosed grade 4 GBM patients in the Danish Glioblastoma Cohort (2016-2019) undergoing whole-exome sequencing. Of 110 patients, 108 (98.2%) consented. Preferences for incidental findings varied: 31% chose no information, 22% actionable findings only, and 42% all information. Despite the complexity, participation was high, suggesting that GBM patients are willing and able to participate in genomic research when flexible consent options are offered.

Glioblastoma (GBM) is the most common malignant brain tumor in adults, with a dismal prognosis despite comprehensive standard treatment consisting of resection and chemo/radiotherapy.^1^ Participation in clinical trials is prioritized to access novel therapies, but enrollment is often restricted to relapse, where many patients are excluded due to declining clinical condition. GBM patients are also often excluded from master protocols, as brain malignancy is commonly an exclusion criterion due to concerns about impaired mental capacity. GBM is associated with cognitive impairment, which may result from the disease itself, perioperative morbidity, and other contributing factors. These impairments may limit a patient’s ability to fully comprehend complex study information.^2^

Patient information, in modern trials, has become increasingly complex. In addition to describing the therapeutic intervention, trial information often includes comprehensive genomic analyses and mandatory statements about incidental findings. Incidental findings (IF) are potential abnormalities that are unrelated to the clinical question for which the test was initiated.^3^ According to Danish legislation, patients must be informed about the possibility of detecting genetic variants that may have implications for their own health or that of their family members. These IF range from variants of uncertain significance to pathogenic germline alterations in genes such as mismatch repair (MMR) or BRCA1/2, which may predispose carriers to secondary cancers or hereditary cancer syndromes. IF have been reported in 1%-18% of cancers with pathogenic variants most common in mesothelioma, ovarian-, cervical-and urothelial cancer, and cancer of unknown primary origin.^4^ The prevalence of IFs in our cohort was 2.2%.^5^

Concerns have therefore been raised that the inclusion of detailed information about genomic testing and incidental findings may act as a barrier to trial participation. Against this background, we investigated whether GBM patients were interested in participating in a comprehensive genomic study and how they expressed preferences regarding the amount of information they wished to receive about IF.

## Methods

We analyzed informed consent forms from the Danish Glioblastoma Cohort.^6^ In the period of 2016-2019 the study enrolled 108 newly diagnosed patients, all of whom were diagnosed as glioblastoma, grade 4.^7^ In 2021, an updated classification was published with a separation of astrocytoma grade 4 into glioblastoma, IDH-wildtype and astrocytoma, IDH mutated, grade 4.^8^ Hence, according to the newest classification, 103 patients had GBM, WHO grade 4, and five patients had astrocytoma, IDH-mutant, WHO grade 4. Whole exome- and RNA sequencing were performed with the purpose of inclusion in targeted experimental trials. The informed consent documents were developed in accordance with national legislation and approved by the Danish ethics committee. Notably, they included a section on incidental findings, where patients indicated their preferred level of information by choosing one of three options:

1. No information–patients would not receive any information about IF.
2. Actionable information only–patients would be informed if IF were clinically actionable, such as when treatment options or preventive measures were available.
3. All information–patients would be informed about any IF, regardless of whether preventive or therapeutic interventions existed.

We were, however, obligated to report findings in a few specific high-risk pathogenic genes (eg *BRCA*).

Consent forms were subsequently reviewed for completeness and patient choice. Forms with missing responses or multiple contradictory marks were categorized as non-assessable.

## Results

Of the 110 patients approached, 108 (98.2%) provided valid consent for participation (Table 1). Two patients (1.9%) declined; one due to limited Danish literacy and one for personal reasons. They were comparable to the rest of the cohort.

**Table 1.** Patient characteristics

Number of patients and %	108
Sex	
Male	44 (41)
Female	64 (59)
Age at diagnosis, median (range)	62 (18-89)
PS	
0-2	106 (98)
>2	2 (2)
MGMT-methylated	48 (44,4)
Tx	
RT/TMZ and adj TMZ	83 (77)
RT +/- TMZ +/-IT (trial)^a^	10 (10)
TMZ monotherapy	2 (2)
60 Gy/30F	5 (5)
34 Gy/10F	7 (7)
None	1 (1)
PFS, median (months)	7.8
OS, median (months)	16.3

PS (Performance status)1 month.

Abbreviations: adj, adjuvant; IT, Immunotherap; OS, overall survival; PSF, progression free survival; RT, radiotherapy; TMX, Temzolodomide; Tx, treatment.

a Checkmate498 + Checkmate548.^9^^,^^10^

Preferences regarding incidental findings varied. Thirty-three patients (30.6%) opted not to receive any incidental findings. Twenty-four patients (22.2%) preferred to be informed only if incidental findings were actionable, while 45 patients (41.7%) marked all findings, even if no preventive or therapeutic options were available. Six consent forms (5.6%) were non-assessable due to missing or multiple selections.

During the course of the study, no pathogenic incidental germline variants were identified in this cohort.

## Discussion

This analysis demonstrates that patients with grade 4 gliomas showed a strong willingness to participate in a comprehensive genomic study, despite the complexity of the information provided and potential cognitive impairment associated with the disease. Nearly all patients provided consent, indicating that concerns about limited comprehension or informational overload may not translate into lower trial participation. No pathogenic incidental germline variants were identified in this cohort; however, actionable mutations have been reported previously.^6^

The distribution of preferences regarding incidental findings highlights the heterogeneity of patient attitudes. Approximately one-third of patients preferred not to receive any information, reflecting a wish to avoid potentially anxiety-inducing or burdensome knowledge. Conversely, over 40% wished to receive all possible information, regardless of actionability. This majority preference underscores a demand for transparency and autonomy, with patients desiring maximal access to information about their health and genetic risks. The intermediate group, representing around 22% of patients, indicates that some individuals value a balance between empowerment and protection, preferring disclosure only when concrete medical interventions are available.

The high participation rate, coupled with the proportion of patients who did not wish to receive any information, also suggests a strong altruistic motivation indicates, despite their own challenging circumstances.

Our findings carry several implications. First, they demonstrate that the inclusion of detailed information about incidental findings does not appear to hinder trial participation. On the contrary, patients and families appear willing to engage with complex genomic research. Finally, while this study was conducted in a Danish setting with specific legal requirements, the findings are broadly relevant as genomic analyses become increasingly integrated into clinical and research protocols worldwide.

The study has some limitations. The assessment was based solely on written consent forms, and we did not conduct qualitative interviews to explore patients’ underlying motivations or understanding. No formal assessment of cognitive function or mental capacity was conducted. This limits our ability to fully account for the extent to which family members may have influenced decision-making or to assess patients’ understanding of the implications of participation and the IF.

## Data Availability

Data will be made available upon reasonable request and should follow Danish legislation.
